# Iris Image Compression Using Deep Convolutional Neural Networks

**DOI:** 10.3390/s22072698

**Published:** 2022-03-31

**Authors:** Ehsaneddin Jalilian, Heinz Hofbauer, Andreas Uhl

**Affiliations:** Department of Computer Science, University of Salzburg, Jakob-Haringer-Straße 2, 5020 Salzburg, Austria; hhofbaue@cs.sbg.ac.at (H.H.); uhl@cs.sbg.ac.at (A.U.)

**Keywords:** deep learning, iris compression, iris recognition

## Abstract

Compression is a way of encoding digital data so that it takes up less storage and requires less network bandwidth to be transmitted, which is currently an imperative need for iris recognition systems due to the large amounts of data involved, while deep neural networks trained as image auto-encoders have recently emerged a promising direction for advancing the state-of-the-art in image compression, yet the generalizability of these schemes to preserve the unique biometric traits has been questioned when utilized in the corresponding recognition systems. For the first time, we thoroughly investigate the compression effectiveness of DSSLIC, a deep-learning-based image compression model specifically well suited for iris data compression, along with an additional deep-learning based lossy image compression technique. In particular, we relate Full-Reference image quality as measured in terms of Multi-scale Structural Similarity Index (MS-SSIM) and Local Feature Based Visual Security (LFBVS), as well as No-Reference images quality as measured in terms of the Blind Reference-less Image Spatial Quality Evaluator (BRISQUE), to the recognition scores as obtained by a set of concrete recognition systems. We further compare the DSSLIC model performance against several state-of-the-art (non-learning-based) lossy image compression techniques including: the ISO standard JPEG2000, JPEG, H.265 derivate BPG, HEVC, VCC, and AV1 to figure out the most suited compression algorithm which can be used for this purpose. The experimental results show superior compression and promising recognition performance of the model over all other techniques on different iris databases.

## 1. Introduction

Biometric human recognition systems are extensively used, and yet are increasing in demand for various applications in recent years. Among all biological traits used, iris is well suited for the most accurate and secure personal identification/verification because of the distinctive patterns present in the iris textures of individuals. To maintain high level of accuracy, the iris images presented to the iris recognition systems need to possess a relentless virtual quality and perception. Nonetheless, considering interoperability and vendor neutrality, often authorities, regulatory bodies, and international standards organizations specify that biometric data must be stored and preserved in image form, rather than in (or in addition to) extracted templates that may depend on proprietary algorithms. Recording raw (high quality) image data in such systems also allows them to benefit from inevitable future improvements in recognition algorithms. However, storage, processing and transmission of such high quality data comes at high cost. To this extent, efficient storage and rapid transmission of iris biometric records is a driving implementation factor in iris recognition system development (especially on low-powered mobile sensors and for portable devices) currently. Image compression techniques reduce the amount of memory used by reducing the number of bits without losing the important data. Image compression will also reduce the transmission time as the transmission time of any image is directly proportional to size of the image. There are two types of compression algorithms, namely “lossless compression” and “lossy compression”. A lossless compression is a reversible process in which no information is lost, but the compression ratio is lower. It is mainly used in the domain where reliability is important: for example executable files and medical data. Lossy compression is a non-reversible process and some information may be lost, but compression ratio is very high and is mainly used in applications where loss of data is acceptable to a certain degree.

Recent machine learning techniques proposed for lossy image compression have generated considerable interest in both the machine learning and image processing communities. Like all lossy compression methods, such models operate on a simple principle: an image, typically proposed as a vector of pixel intensities, is quantized, reducing the amount of information required to record or transmit it, which incorporates introducing some error at the same time. Typically, it is not the pixel intensities that are quantized directly. Rather, an alternative (latent) representation of the image is found, and quantization takes place in this representation, yielding a discrete-valued vector. To be more precise, deep learning models learn (extract) the key features in the input image through back propagation training and enable the compression of image information, preserving and restoring such features without too much prior knowledge.

In this work, we extend our previous study [1] on evaluating the expediency of a Deep Semantic Segmentation-based Layered image Compression (DSSLIC) model [2] for iris compression within a biometric recognition framework. As a distinction to the previous work: We utilize a more powerful deep-learning-based model (End-to-end Optimized Image Compression model (EOIC)) to address the lower performance issue of the Conditional Probability Models for Deep Image Compression (CPDIC), as used as the comparing model in the previous experiments. We further consider three new state-of-the-art (non-learning-based, lossy) image compression models along with a couple of other commonly used image compression algorithms to compress iris images in four well-known iris datasets. To evaluate the compression performance in each case, in addition to the Multi-scale Structural Similarity Index (which was used in our previous work), another “Full-Reference” image quality assessment measure, namely Local Feature Based Visual Security (LFBVS), and a “No-Reference” images quality assessment measure, namely Blind Reference-less Image Spatial Quality Evaluator (BRISQUE) are used. The biometric recognition performance then is evaluated, in terms of Equal Error Rate (EER), by using the compressed iris images in some predefined iris biometric systems. At the end, the compression and the corresponding recognition results are compared and carefully analyzed to figure out a well suited compression algorithm to be employed in iris recognition systems. The rest of the paper is structured as follows: Section 2 will review related works and state of the art, and Section 3 will describe the deep-learning based model (DSSLIC). Section 4 will give the details of the experimental framework, and the experiments and analysis will be presented in Section 5. Finally, Section 6 will conclude the paper.

## 2. Related Work

Many studies have been conducted on iris image compression and the subsequent recognition performance during the past decades). For example: Matschitsch et al. [3] studied the impact of applying different lossy compression algorithms on the matching accuracy of iris recognition systems, relating rate-distortion performance to the matching scores. The authors in this work concluded that JPEG2000, SPIHT, and PRVQ are almost equally well suited for iris compression. Daugman and Downing [4] analyzed the effect of severe image compression on iris recognition performance and introduced a schemes that combine region-of-interest isolation with JPEG and JPEG2000 compression at severe levels, and tested them using a publicly available database of iris images. Grother [5] investigated existing image compression approaches and compared JPEG and JPEG2000 to provide a quantitative support to the revision of the ISO/IEC IS 19794-6, considering: cropped format (IREX K3), masked and cropped format (IREX K7), and an unsegmented polar format (IREX K16) in his experiments. Ives et al. [6] investigated the effects of image compression on recognition system performance using a commercial version of the Daugman “iris2pi” algorithm along with JPEG-2000 compression, and linked that to image quality. Korvath et al. [7] evaluated the impact of dedicated lossless image codecs (lossless JPEG, JPEG-LS, PNG, and GIF), lossless variants of lossy codecs (JPEG2000, JPEG XR, and SPIHT), and some general purpose file compression schemes on the iris images. Bergmüller et al. [8] studied the impact of using pre-compressed data in iris segmentation and evaluated the relation between iris segmentation performance and general image quality metrics. Rathgeb et al. [9] investigated the impact of image compression on iris segmentation algorithms. In their work, they examined the impact of severe image compression using in particular, JPEG, JPEG 2000, and JPEG-XR algorithms on the performance of different iris segmentation approaches.

With recent advancements in deep learning techniques, researchers have proposed few learning based image compression methods as well. Toderici et al. [10] used a recurrent neural networks, based on convolution and deconvolution long short-term memory (LSTM), to extract binary representations which are later compressed with entropy coding. Ballé et al. [11] proposed a compression framework that included a generalized divisive normalization (GDN)-based nonlinear analysis transform, a uniform quantizer, and a nonlinear synthesis transform. Theis et al. [12] introduced a deep-learning-based auto-encoder in which they used smooth approximation instead of quantization to obtain different rates. Agustsson et al. [13] used a soft-to-hard vector quantization model along with a unified formulation for both the compression of deep learning models and image compression. Jiang et al. [14] utilized a compact convolutional neural network (ComCNN) and a reconstruction convolutional neural network (RecCNN) to encode and decode the original image, respectively. Johnston et al. [15] utilized the structural similarity (SSIM) quality measure and spatially adaptive bit allocation to further improve the performance. Li et al. [16] introduced a model which was based on image content weighting. They used the edge feature maps, extracted by a convolution neural network, as the importance map of the original image. In this work, an innovative algorithm was introduced to solve the non-differentiated calculation in the quantization rounding function to achieve a backward propagation gradient in the standard image algorithm. Luo et al. [17] used the benefit of image compression and classification to reconstruct the images and to generate corresponding semantic representations simultaneously. Mantzer et al. [18] proposed a conditional probability model for deep image compression (CPDIC), with concentration on improving the entropy rate of the latent image representation using a context model (a 3D-CNN which learns a conditional probability model of the latent distribution). During training the auto-encoder makes use of the context model to estimate the entropy of its representation, and the context model is concurrently updated to learn the dependencies between the symbols in the latent representation. Wang et al. [19] proposed a compression bit allocation algorithm, which allows a recurrent neural network (RNN)-based compression network to hierarchically compress the images according to semantic importance maps.

**Figure 1:**
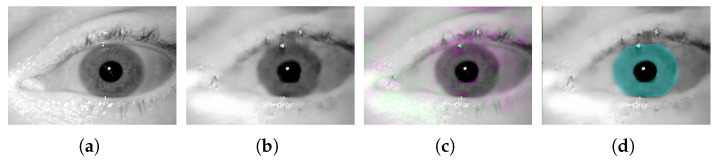
An iris image (**a**) and its corresponding output using a GAN based model (**b**), along with an image visualizing their difference (**c**), and the overlaid ground-truth (**d**).

Some other works utilized Generative Adversarial networks (GAN) in their architecture to learn the image compression. Santurkar et al. [20] utilized a discriminator to help training of a compression decoder. They calculated a perceptual loss, which was based on the feature map obtained from pretrained ImageNet and AlexNet. Only low-resolution image coding results were reported in this work. Ripple et al. [21] embedded an auto-encoder in a GAN architecture, where the feature extraction adopted a pyramid of inter-scale alignments. They considered the target and its reconstruction jointly as a single entity and, instead of producing an output for classification at the last layers of the network, accumulated scalar outputs along branches constructed at different roots. The average of these scalars was used as the final value passed to a sigmoid function. Augustesson et al. [22] proposed an image compression (synthesis) model based on a GAN architecture. They consider two modes of operations namely “generative compression” and “selective generative compression”. The later approach generated parts of image from a semantic label map. Their model was concentrated on obtaining extremely low bit-rates rather than on preserving key features in image, and to this extent a clear trade off between these two factors remained unclear. Balle et al. [23] proposed an end-to-end trainable encoder–decoder framework for image compression. The encoder part includes a variational generative model, whose out put is quantized, compressed, and transmitted as side information. The decoder recovers the quantized vector from the compressed signal. It then obtains the correct probability estimates to successfully recover and reconstruct the image. Minnen et al. [24] introduced a model which jointly optimizes an autoregressive component that predicts latents from their causal context (Context Model) along with a hyperprior and the underlying autoencoder. Real-valued latent representations then are quantized to create integer-valued latents and hyperlatents, which are compressed into a bit-stream using an arithmetic encoder and decompressed by an arithmetic decoder. Cheng et al. [25] used discretized Gaussian Mixture Likelihoods to parameterize the distributions of latent codes, which removes redundancy to achieve an accurate entropy model, and leads to fewer encoding bits. They take advantage of attention modules to make the learned models pay more attention to complex regions. In any case, the main drawback of applying GAN networks (at least on their own and without further information fusion) to reconstruct the images is their inability to preserve key fine features in the images reconstructed. This phenomenon is observable in the corresponding results obtained using the above models too. In fact, this issue can directly affect the iris key traits and their subsequent recognition performance in the reconstructed images. Figure 1 shows a sample iris image (Figure 1a) (from the Notredame iris dataset, as used in this work), and its compressed version (Figure 1b), using the last GAN based model, along with their difference image (Figure 1c), and the overlaid ground-truth mask (Figure 1d). Gray regions in the difference image show where the two images have the same intensities, and magenta and green regions show where the intensities are different. Furthermore, the overlaid ground-truth mask shows how the actual iris outer and inner boundaries (as specified by the mask) are distorted in the compressed (reconstructed) image. Nevertheless, while the recent architectures (e.g., et al. [23,24,25]) contributed to improve the compressive performance in one way or another, yet their experimental trail (carried out in this research work) revealed that such improvement has come at the cost of loosing some level of scalability. In particular, during our experiments we noticed that on certain data (e.g., Casia5a as used in our experiments), these models are only able to preserve and reconstruct key image features when higher bits rates are used, and simply do not converge to lower bit rates even when highest compression parameters are used. To this extent, we excluded them from our comparison experiments.

## 3. Deep-Learning-Based Image Compression Model (DSSLIC)

**Figure 2:**
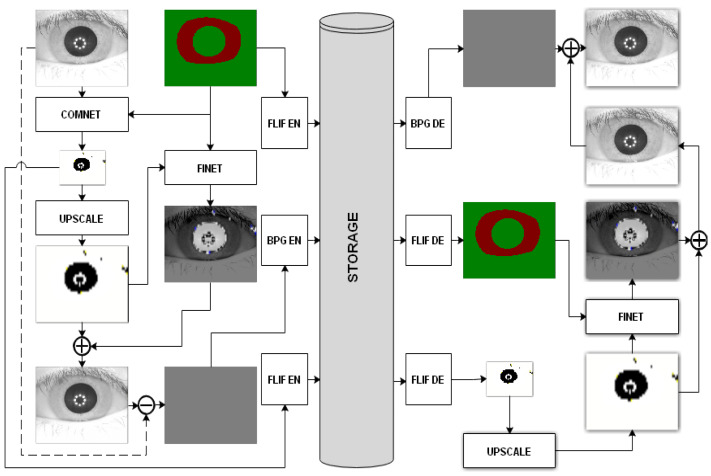
The deep-learning-based iris compression model.

Figure 2 demonstrates the overall architecture of the model used in our work, which is derived from the DSSLIC model already introduced in [2]. As a key distinction to the original model, here we do not use a segmentation network in our model, and instead we provide the manually segmented labels directly to the model. Doing so, we try to provide a better evaluation of the actual performance of the model by introducing more accurate labeling data. In fact, such a configuration is practically justifiable as the recent advancements in the deep-learning-based segmentation techniques has made highly accurate segmentations (that are comparable to the manually segmented labels) available in a timely manner (i.e., [26]).

The overall architecture of the model is composed of an encoding part and a decoding part. The encoding part includes two deep learning networks, namely “CompNet” and “FiNet” (a GAN-based network). The ComNet network takes the iris image as input, while the corresponding segmentation map is encoded to serve as side information to this network for generating a low-dimensional version of the original image. Both the segmentation map and the compact version are losslessly encoded using the FLIF codec [27], which is a state-of-the-art lossless image compression algorithm. Having the segmentation map and up-sampled compact image, the FiNet works to obtain a high-quality reconstruction of the input image. Unlike the similar architectures in which the GAN networks operate directly only on the input images, here in this model, the GAN network takes the segmentation map as the input and tries to learn the missing detail information of the up-sampled version of the compacted input image to minimize the distortion of the synthesized images. It should be noted that although GAN-based synthesized images generated with the help of the segmentation maps are visually appealing, their details can be quite different from the original images. To minimize the errors of the synthesized images, the up-sampled version of the compact image, as an additional input, is used. By doing so, the FiNet learns the missing detail information of the up-sampled version of a compact image with respect to the input image, which in turn controls the output of the GAN network. After adding the up-sampled version of the compact image and the FiNet’s output, we obtain a better estimate of the input. The residual difference between the input and the estimate is then obtained and encoded by a lossy codec (H.265/HEVC intra coding-based BPG). In order to deal with negative values, the residual image is re-scaled to [0, 255] with a min-max normalization before encoding. The min and max values are also sent to the decoder for inverse scaling. In this scheme the segmentation map serves as the base layer and the compact image and the residual are the first and second enhancement layers, respectively. At the decoder side the segmentation map and the compact representation are decoded to be used by the FiNet to obtain an estimate of the input image. The output of FiNet is then added to the decoded residual image to obtain the reconstructed image as output.

## 4. Experimental Framework

In this section, we describe the methodological details of our experiments including: the databases used, the comparison compression algorithms, metrics and measures, and the recognition pipeline.

### 4.1. Datasets

For our experiments, we used four different iris datasets: The Notredame dataset [28] includes 835 iris images of 30 different subjects. The images in this dataset are taken in near-infrared spectrum in an indoor environment with the LG 2200 iris biometric system. The Casia4i dataset [29] includes 2640 iris images of 249 subjects. Images in this dataset are acquired under near-infrared illumination, with a close-up iris camera. The IITD dataset [30] includes 2240 iris images of 224 subjects. The images are acquired in the indoor environment, with the Jiris, Jpc1000 digital CMOS camera in near-infrared spectrum. The Casia5a dataset [29] includes 1880 images of both eyes of 94 users. The dataset comprises images captured from a video sequences taken in 2009 and 2013. Special attention should be paid to the fact that: the selection of the datasets and the number of samples included in each case were subject to the availability of the ground-truth masks which were required for the training and the segmentation process. The ground-truth masks were provided by the Multimedia Signal Processing and Security Lab (WaveLab) group at the University of Salzburg.

### 4.2. Comparing Compression Algorithms

To evaluate the model expediency, primarily we compare its performance against a well-known and popular deep-learning based (lossy) compression algorithm. End-to-end Optimized Image Compression model (EOIC) [11] consists a nonlinear analysis transform, a nonlinear synthesis transform and a uniform quantizer. Each transform begins with an affine convolution on the *i*th input channel *u* of the *k*th stage at spatial location (m,n):

(1) $$v_{i}^{\left(k\right)}\left(m,n\right)=\sum_{j}\left(h_{k,ij}*u_{j}^{\left(k\right)}\right)\left(m,n\right)+c_{k,i},$$

followed by a down-sampling:

(2) $$w_{i}^{\left(k\right)}\left(m,n\right)=v_{i}^{\left(k\right)}\left(s_{k}m,s_{k}n\right),$$

where $s_{k}$ is the down-sampling factor for stage *k*. Each stage concludes with a Generalized Divisive Normalization (GDN) transform:

(3) $$u_{i}^{\left(k+1\right)}\left(m,n\right)=\frac{w_{i}^{\left(k\right)}\left(m,n\right)}{{\left(\beta_{k,i}+\sum_{j}\gamma_{k,ij}{\left(w_{j}^{\left(k\right)}\left(m,n\right)\right)}^{2}\right)}^{\frac{1}{2}}}.$$

The full set of *h*, *c*, β, and γ parameters (across all the three stages) constitute the optimization parameters. This representation then is quantized, yielding a discrete-valued vector which is then compressed. The rate of this discrete code is lower-bounded by the entropy of the discrete probability distribution of the quantized vector. To reconstruct the compressed image, the discrete elements of the vector are reinterpreted as a continuous-valued vector which are transformed back to the data space using a parametric synthesis transform. Analogously, the synthesis transform consists of three stages, with the order of operations reversed within each stage, down-sampling replaced by up-sampling, and GDN replaced by an approximate inverse called IGDN:

(4) $${\hat{w}}_{i}^{\left(k\right)}\left(m,n\right)={\hat{u}}_{i}^{\left(k\right)}\left(m,n\right)\cdot {\left({\hat{\beta }}_{k,i}+\sum_{j}{\hat{\gamma }}_{k,ij}{\left({\hat{u}}_{j}^{\left(k\right)}\left(m,n\right)\right)}^{2}\right)}^{\frac{1}{2}}.$$

which is followed by up-sampling:

(5) $${\hat{v}}_{i}^{\left(k\right)}\left(m,n\right)={\hat{w}}_{i}^{\left(k\right)}\left(m/{\hat{s}}_{k},n/{\hat{s}}_{k}\right),$$

where ${\hat{s}}_{k}$ is the up-sampling factor for stage *k*. Finally, this is followed by an affine convolution:

(6) $${\hat{u}}_{i}^{\left(k+1\right)}\left(m,n\right)=\sum_{j}\left({\hat{h}}_{k,ij}*{\hat{v}}_{j}^{\left(k\right)}\right)\left(m,n\right)+{\hat{c}}_{k,i}.$$

Analogously, the set of $\hat{h}$, $\hat{c}$, $\hat{\beta }$, and $\hat{\gamma }$ make up the parameter vector θ. We further compare the model performance against some of the most popular and state-of-the-art non-learning-based (lossy) compression methods including: JPEG [31], the current ISO standard JPEG2000 (J2K) [31], the H.265 derived BPG [32], HEVC [33] (HM version 16.20) [34], VVC [35] (VTM version [36] and AV1 [37] (version 2.0) [38] algorithms.

### 4.3. Metrics and Measures

To evaluate the compression performance we considered three different measures, including two Full-Reference (FR) image quality assessment measures and one objective blind or No-Reference (NR) image quality assessment measure. Full-Reference (FR) image quality assessment requires as input not only the compressed image, but also a “clean”, pristine reference image with respect to which the quality of the compressed image is assessed. Objective blind or No-Reference image quality assessment requires as input only the compressed image, and assesses the quality of the compressed image objectively. Multi-Scale Structural Similarity Index Measure (MS-SSIM) is the primary Full-reference (FR) image quality assessment measure we considered in our experiments. Unlike in Structural Similarity Index Measure (SSIM), where variation in luminance, contrast and structure of “single-scale” input images are compared, MS-SSIM repeatedly down-samples the input images up to *M* scales. At each scale, the contrast comparison and the structure comparison are calculated. The luminescence comparison is computed only at scale *M*, and the final MS-SSIM evaluation is obtained by combining the measurements at different scales [39]. The value of the MS-SSIM measure is bounded between 0 and 1, where 0 represents the worst quality and 1 the best quality. Local Feature Based Visual Security (LFBVS) [40] is the second Full-reference (FR) image quality assessment measure we considered in our experiments. The algorithm utilizes localized edge and luminance features which are combined and weighted according to error magnitude, i.e., error pooling. The value of the LFBVS measure is bounded between 0 and 1, where 0 represents the best quality and 1 the worst quality. We further used Blind/Reference-less Image Spatial Quality Evaluator (BRISQUE) [41] as our No-Reference image quality assessment measure. BRISQUE does not compute distortion-specific features, such as ringing, blur, or blocking, but instead uses scene statistics of locally normalized luminance coefficients to quantify possible losses of “naturalness” in the image due to the presence of distortions, thereby leading to a holistic measure of quality. The underlying features used derive from the empirical distribution of locally normalized luminances and products of locally normalized luminances under a spatial natural scene statistic model. The value of the BRISQUE measure is bounded between 0 and 100, where 0 represents the best quality and 100 the worst quality. In the recognition experiments, the Equal Error Rate (EER) was chosen as an overall measure of biometric recognition performance. EER is the operation point on the receiver operating characteristic curve where the false non-match rate and the false match rate are equal.

### 4.4. Recognition Pipeline

In our recognition pipeline, we used the contrast adjusted Hough transform (CAHT) [42], and Osiris [43] for iris segmentation, local Gabor filters (LG) for feature extraction, and the Hamming distance with rotation correction for matching. Apart from the Osiris, we used the implementations provided in the USIT toolkit of the University of Salzburg [44].

## 5. Experiments and Analysis

**Figure 3:**
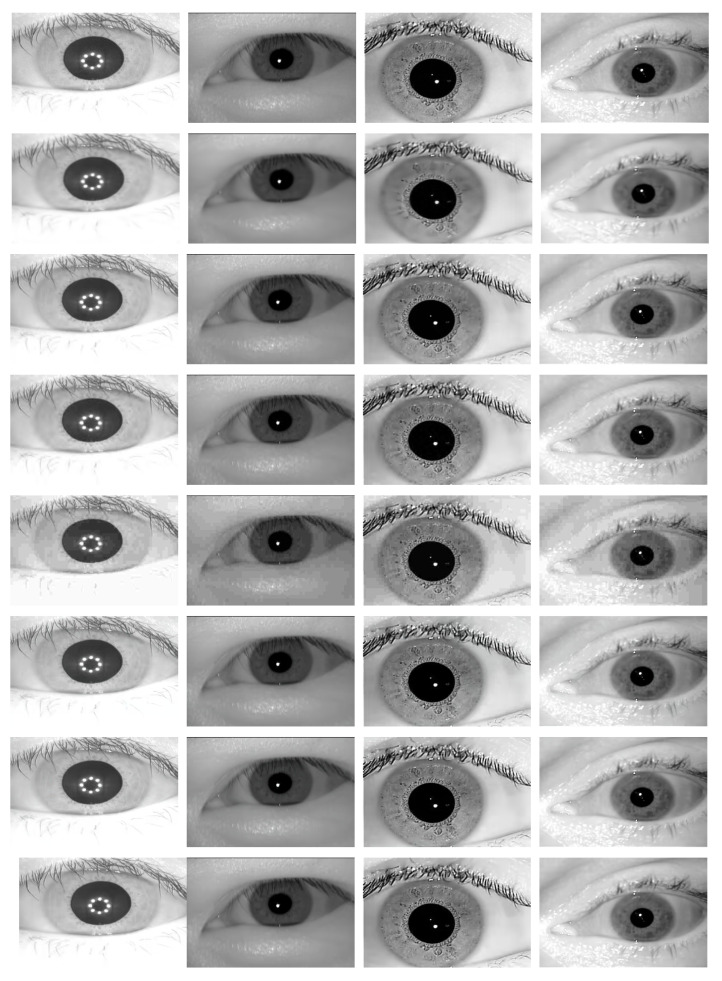
Samples of highly (A) compressed iris images from the Casia4i, Casia5a, IITD, and Notredame datasets, per column, respectively, using DSSLIC, EOIC, BPG, J2K, JPEG, HEVC, VVC, and AV1 algorithms per row, respectively.

Addressing the input size requirement of the deep-learning-based models and also a fair evaluation policy, we rescaled all the images in our datasets to the size: 256×512 during the compression evaluation experiments. Since the networks are trained on RGB format we cloned each image two times to generate 3 channel (RGB) images (256×512×3). We applied a cross-fold scheme to train the (deep-learning-based) models. For this, first we partitioned each dataset into two equal parts and trained the models on one partition and tested it on the other partition. We switched the partitions role next, and doing so we tested the networks on all samples in each dataset without overlapping the training and testing sets. For the DSSLIC model, we set the down-scaling factor α=8 to obtain the compact representation of the inputs. Table 1 summarizes the training parameters used for each model. Likewise, we applied the other comparison (non-learning-based) compression algorithms to our datasets. To address the preset bandwidth/storage compression limit requirement we defined two bandwidth limits of 0.30 (A) and 0.60 (B), corresponding to the higher and the lower compression levels, respectively, for each dataset in terms of bit-per-pixel (bpp). Obviously, not all algorithms allow to set the exact output file size. Thus, we set the compression parameter for each algorithm in way that the achieved bpp of the resulting compressed images are equal to or less than the predefined bandwidth/storage limit. It is also important to note that the resulting file sizes using the DSSLIC model are among the smallest in the majority of cases (i.e., Casia4i, Casia5a-A, IITD-B, and Notredame). Table 2 shows the selected compression parameters (par) and the resulting bpps per algorithm and dataset. Furthermore, samples of the output (compressed) images in each dataset using the compression methods used are presented in Figure 3 per column and row, respectively.

**Figure 4:**
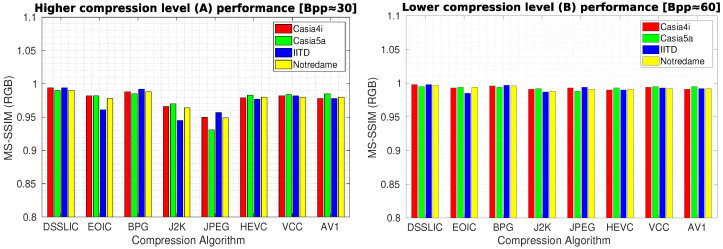
Compression performance in terms of MS-SSIM for the high (**left** graph) and low (**right** graph) compression levels.

Table 3 presents the quality compression results in terms of MS-SSIM measure for each dataset (averaged over all images) using the different compression algorithms. As it can be seen, the DSSLIC model shows superior performance over all other algorithms for both compression levels considered. This is a quite interesting result given the fact that the files produced by the DSSLIC are among the smallest files produced by the methods applied. Visual inspection of the obtained output iris images as presented in Figure 3 (the first row) also shows that the model is able to preserve the key iris biometric traits and the structural features very well. Across all datasets, and both compression settings, BPG is (almost) always the second-best. The performance of the other six algorithms vary depending on the datasets: The VCC algorithm shows relatively better performance (compared to the other five algorithms) on the Casia4i, Notredame, and IITD datasets (only when the higher compression rate (A) is considered), while the AV1 algorithm demonstrates overall a better performance than VCC on the Casia5a dataset. EOIC performs better (than VCC and AV1) on Notredame datasets (only when the lower compression rate (B) is considered). The other algorithms (J2K, HEVC, and JPEG) come thereafter while their performance in the majority of cases is lower than the other algorithms across different datasets. For the sake of better interpretation, we visualized the corresponding performance in the form of bar-graphs (for each compression level) in Figure 4.

**Figure 5:**
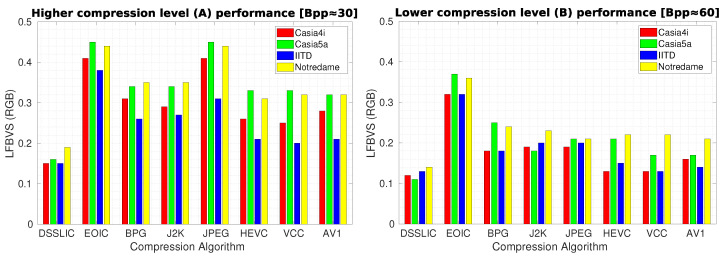
Compression performance in terms of LFBVS for the high (**left** graph) and low (**right** graph) compression levels.

Table 4 shows the corresponding results for each dataset (averaged over all images) in terms of LFBVS. The superior performance of DSSLIC over the other algorithms is visible in these results too. However, when it comes to the other algorithms’ performance, the MS-SSIM experiment rankings do not apply any more. All together HEVC, VCC, and AV1, rank the second best, showing very close performance to each other. Among the other four algorithms: BPG and J2K come thereafter showing better performance than JPEG in the majority of the cases. Yet, their performance seems to vary depending on the compression level applied and across different datasets. For instance, BPG shows better performance (than J2K) when considering IITD dataset or the lower compression rate (B) on the Casia4i dataset, while J2K shows better or at least equal performance in all other cases. At the end comes EOIC which shows the worst performance almost in all cases. Figure 5 demonstrates the algorithms’ performance for each compression level in the form of bar-graphs.

**Figure 6:**
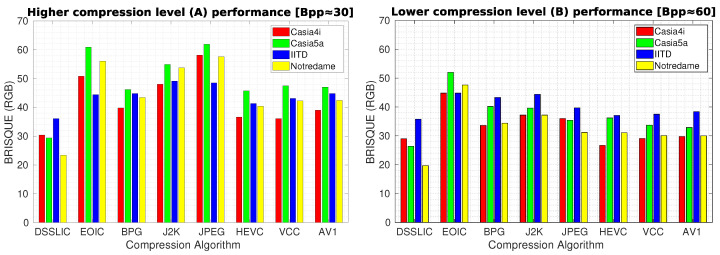
Compression performance in terms of BRISQUE for the high (**left** graph) and low (**right** graph) compression levels.

In order to provide an objective insight into the quality of images generated, we calculated the BRISQUE measure for the obtained compressed images. Table 5 shows the corresponding results for each dataset (averaged over all images) in terms of BRISQUE. The dominating performance of DSSLIC over the other algorithms is visible in these results also. When it comes to the other algorithms’ performance, the LFBVS experiment performance rankings rather applies: EOIC again shows the worst performance almost in all the cases, and all together HEVC, VCC, and AV1, rank the second best, showing very close performance to each other. Among the other three algorithms: BPG comes next showing better performance than the other two algorithms (J2K and JPEG) in the majority of the cases (excluding the Lower compression level (B) for the Casia5a, Notredame, and IITD datasets), and J2K and JPEG together come at the end, showing rather similar performance. Figure 6 demonstrates the algorithms’ performance for each compression level in the form of bar-graphs as well. Unsurprisingly, in all the compression evaluation experiments (MS-SSIM, LFBVS, and BRISQUE), the higher compression rate (reflected in the left graphs in the corresponding figures) decreased the compression performance over all the datasets and algorithms (including DSSLIC).

In the next stage of our experiments, we applied the biometric recognition pipeline (as described in Section 4) to all the compressed images obtained, and evaluated the biometric comparison accuracy, in terms of EER, for the two levels of compression. In addition to the two segmentation algorithms (Osiris and CAHT) used, we considered an optimal segmentation configuration as well, utilizing the manually annotated segmentation drop masks. The optimal segmentation configuration was used to disentangle the distorting effect of the compression on the iris unique biometric traits (which directly affect their subsequent recognition performance) from the iris structural feature distortions which may cause the segmentation failures. Table 6 and Table 7 show the recognition results using the CAHT segmentation and manual segmentation, respectively. As it can be seen in Table 6, recognition does not work at all for the Casia5a and Notredame datasets when using the CAHT segmentation algorithm. The DSSLIC compression shows the best performance only on the IITD and Casia4i datasets (when higher compression rate (A) considered). However, when we use the manual segmentations (Table 7): while the recognition still does not work for Notredame data, yet for the remaining datasets, DSSLIC results are never surpassed by any other comparison compression algorithm. Given the fact that the DSSLIC also produces the smallest actual files, these results imply that DSSLIC compression is able to preserve the iris unique biometric traits bests-certainly better than the other comparison algorithms, as the segmentation defects were ruled out using the manual segmentations.

Table 8 demonstrates the results when applying the OSIRIS algorithm for segmentation. As it can be observed, recognition on the Notredame data does not work either, but otherwise the ranking of the algorithms is fairly different. DSSLIC no longer performs best in any case. If we compare the results obtained using CAHT and OSIRIS segmentation algorithms, we can easily interpret that the segmentation methods and the logic behind them can react quite differently to the artifacts introduced to the image during the compression process, and thus deliver very different results having identically compressed iris images as the input. Overall, the clearly higher compression performance of the DSSLIC algorithm is not directly translated into best recognition accuracy, except in the configuration where the manual segmentation is used. It should be noted that by using the manual drop masks, the measures are only impacted by the possible artifacts introduced by the compression to the iris texture, whereas when using CAHT and OSIRIS as the segmentation modules, the measures are impacted by both the eventual segmentation failures introduced by the segmentation modules, as well as the artifacts introduced by the compression.

**Figure 7:**
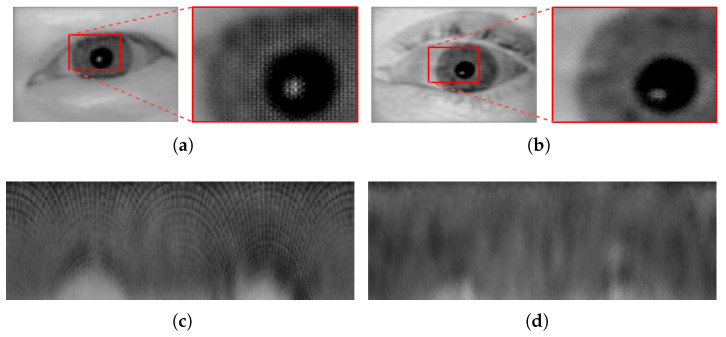
Sample compressed images in the Casia5a dataset (**a**) and Notredame datasets (**b**) and their corresponding normalized iris images (**c**,**d**), respectively, both generated by CPDIC algorithm.

**Figure 8:**
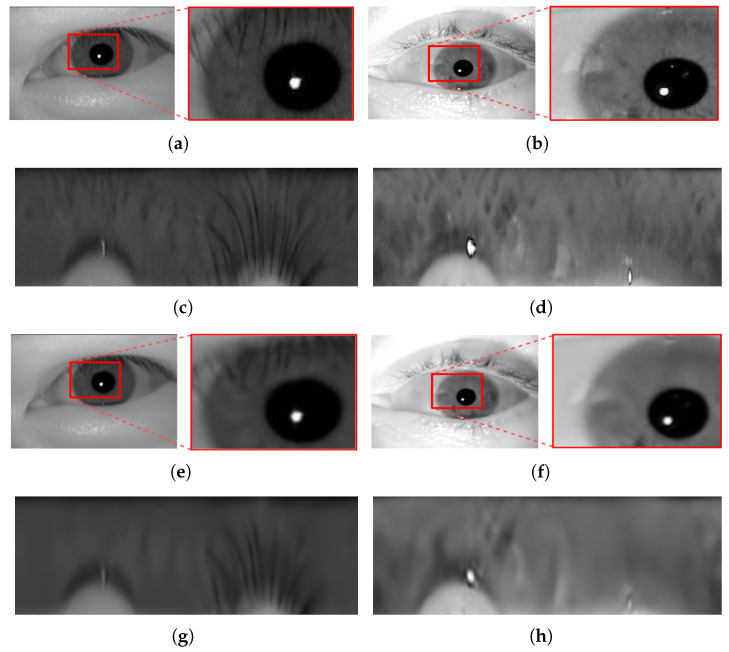
Sample images in the Casia5a (**a**) and Notredame (**b**) datasets, and their corresponding normalized iris images (**c**,**d**), along with their compressed images (**e**,**f**) and their corresponding normalized versions (**g**,**h**), respectively, using EOIC algorithm.

Concerning the other deep-learning-based algorithm (EOIC) performance: The algorithm proved (based on the results obtained) to perform better than the CPDIC model which we used in our previous work [1]. The analysis of the output images generated by the CPDIC model (see Figure 7) in our previous work revealed some artifacts which were distributed uniformly over all the images in a block-shaped pattern. These artifacts were more severe and intense in the high texture areas, specifically the iris texture areas. The persistence of these artifacts over all images clearly undermined their recognition performance. When inspecting the iris images obtained using the EOIC model (see Figure 8), we can clearly observe that such artifacts do not exist, and thus the perceptual quality of the images generated are much better than those generated by CPDIC model. Yet, we should note that the iris unique biometric traits seem not to be well preserved and reconstructed in the generated images compared to the images generated by, e.g., DSSLIC. This in fact directly affects the the corresponding recognition results as reflected in Table 7.

**Figure 9:**
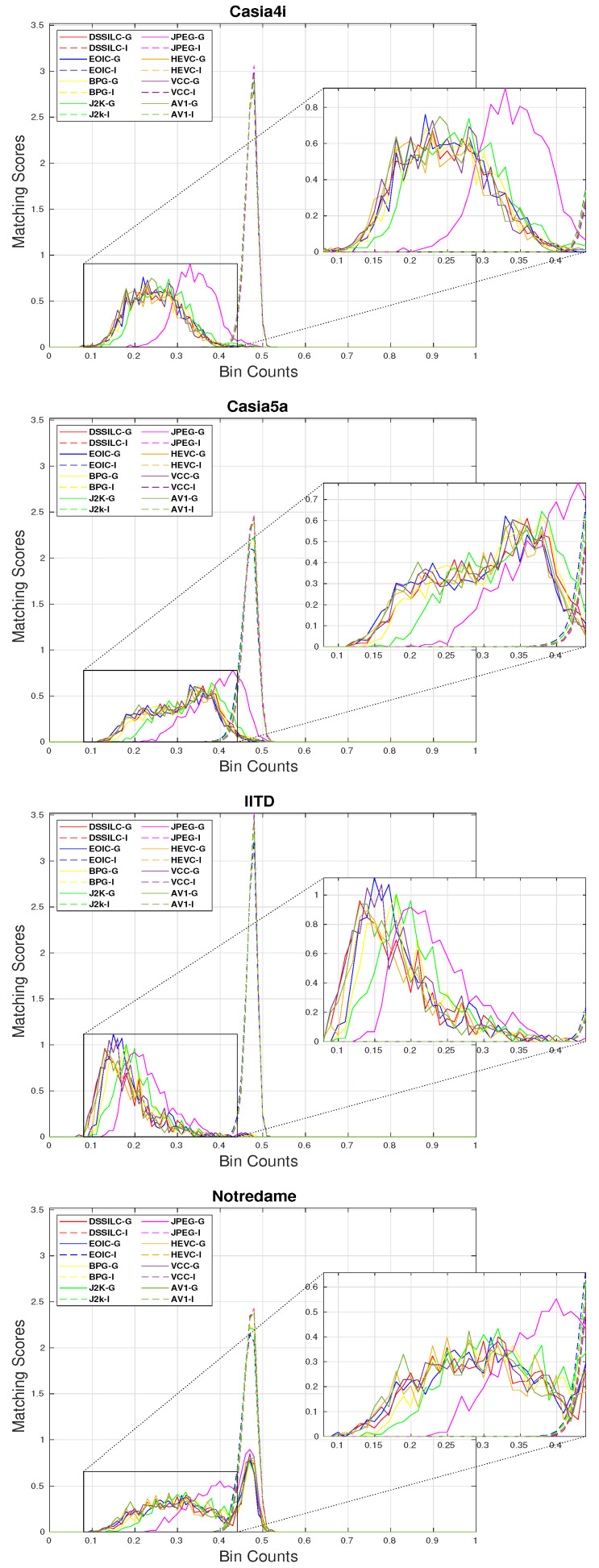
Genuine and impostor distributions of the different compression methods.

We further analyzed the distribution of the genuine and impostor scores obtained during the recognition experiments, to provide a better understanding of how the quality of biometric traits in the compressed images can affect their actual recognition performance. Figure 9 demonstrates the genuine and impostor distributions for the different compression methods for each dataset when considering the optimal segmentation configuration (to exclude the influence of probable segmentation errors). Each pair of curves (genuine and impostor) are indicated by color while line-type distinguishes between impostor (dashed) and genuine (solid). As it can be seen, the impostor curves remain virtually unchanged, while the genuine curves fluctuate almost in all cases. This leads us to the observation that the compression process affects the genuine scores, by introducing artifacts into the iris images which subsequently alter the distinct patterns that are present in the genuine samples, making the compressed images more dissimilar, and thus deteriorating the overall EER scores at the end.

## 6. Conclusions

We investigated the performance of a deep-learning-based image compression model (DSSLIC) along with some other well-known models in terms of rate-distortion and the subsequent recognition accuracy. The model showed superior compression performance over all other algorithms using different datasets and compression rates. Unlike the other algorithms, the DSSLIC model was able to cope with iris images with complex feature characteristics, and possessed stable performance on all different types of iris data. Visual inspection of the iris images obtained using the other comparison deep-learning-based model (EOIC) showed that: while DSSLIC model possesses better preforming profile than the CPDIC (as used in our previous work), yet it is not able to well preserve and reconstruct the iris unique biometric traits in the images, resulting in lower recognition performance (compared to the DSSLIC model). Furthermore, the experiments with different segmentation algorithms revealed that the segmentation technique and the logic used in it could react quite differently to the compressed images, which in the case of, e.g., the Osiris algorithm caused considerable degradation of the segmentation performance. The results obtained using the manual drop masks supported this argument too. To this extent, as the recognition experiments results showed, the higher compression performance of the DSSLIC algorithm was directly translated into better recognition rates only when the affecting role of the segmentation failures were precluded with the help of the manual drop masks. The experiments also showed that an increase in compression results in reduction of recognition performance in the majority of cases. Analysis of the genuine and the impostor scores indicated that compression introduces artifacts into the iris images which alter the iris unique biometric traits that are present in the genuine samples, making the compressed images more dissimilar. Overall, the results showed that the presented deep-learning-based model is capable of efficient iris image compression, and can be used in an iris biometric recognition system efficiently.

## Figures and Tables

**Table 1:** Networks’ training parameters.

Network	DSSLIC	EOIC
Optimizer	Adam	Adam
Learning rate	0.0002	0.0001
Momentum	-	-
Weight decay	0.0005	0.1
Iteration	250	1,000,000

**Table 2:** Selected compression parameters (par) and their corresponding compression performance in bits per pixel (bpp) for each algorithm.

Dataset	Casia4i				Casia5a				IITD				Notredame			
Method	par	bpp	par	bpp	par	bpp	par	bpp	par	bpp	par	bpp	par	bpp	par	bpp
DSSLIC	23	0.20	16	0.44	23	0.16	14	0.45	27	0.30	19	0.53	23	0.16	14	0.51
EOIC	1024	0.21	4096	0.47	1536	0.16	7680	0.43	256	0.28	1792	0.53	512	0.16	5120	0.53
BPG	37	0.21	30	0.54	30	0.19	24	0.42	33	0.29	26	0.60	33	0.18	24	0.55
J2K	35	0.23	21	0.55	45	0.18	14	0.55	28	0.27	14	0.55	45	0.18	14	0.55
JPEG	23	0.20	57	0.50	12	0.19	57	0.51	17	0.30	57	0.53	09	0.18	57	0.58
HEVC	32	0.24	27	0.44	28	0.18	20	0.44	36	0.30	20	0.56	32	0.21	25	0.51
VCC	31	0.23	25	0.48	30	0.16	22	0.51	34	0.31	28	0.57	32	0.17	25	0.51
AV1	31	0.22	25	0.44	30	0.20	13	0.55	42	0.30	30	0.58	35	0.20	20	0.51
Bpp (max)	A (0.30)		B (0.60)		A (0.30)		B (0.60)		A (0.30)		B (0.60)		A (0.30)		B (0.60)	

**Table 3:** Average MS-SSIM scores using high (A) and low (B) compression levels.

Dataset	Casia4i		Casia5a		IITD		Notredame	
Level	B	A	B	A	B	A	B	A
DSSLIC	0.998	0.994	0.995	0.990	0.998	0.998	0.997	0.998
EOIC	0.993	0.982	0.994	0.982	0.985	0.961	0.994	0.978
BPG	0.996	0.988	0.994	0.985	0.997	0.992	0.996	0.988
J2K	0.991	0.966	0.992	0.970	0.987	0.945	0.988	0.964
JPEG	0.993	0.950	0.988	0.931	0.994	0.957	0.991	0.949
HEVC	0.990	0.979	0.993	0.983	0.990	0.977	0.991	0.980
VCC	0.994	0.982	0.995	0.984	0.993	0.982	0.992	0.980
AV1	0.991	0.978	0.995	0.985	0.992	0.978	0.992	0.980

**Table 4:** Average LFBVS scores using high (A) and low (B) compression levels.

Dataset	Casia4i		Casia5a		IITD		Notredame	
Level	B	A	B	A	B	A	B	A
DSSLIC	0.12	0.15	0.11	0.16	0.13	0.15	0.14	0.19
EOIC	0.32	0.41	0.37	0.45	0.32	0.38	0.36	0.44
BPG	0.18	0.31	0.25	0.34	0.18	0.26	0.24	0.35
J2K	0.19	0.29	0.18	0.34	0.20	0.27	0.23	0.35
JPEG	0.19	0.41	0.21	0.45	0.20	0.31	0.21	0.44
HEVC	0.13	0.26	0.21	0.33	0.15	0.21	0.22	0.31
VCC	0.13	0.25	0.17	0.33	0.13	0.20	0.22	0.32
AV1	0.16	0.28	0.17	0.32	0.14	0.21	0.21	0.32

**Table 5:** Average BRISQUE scores using high (A) and low (B) compression levels.

Dataset	Casia4i		Casia5a		IITD		Notredame	
Level	B	A	B	A	B	A	B	A
DSSLIC	29.0	30.4	26.4	29.4	35.8	36.1	19.7	23.4
EOIC	44.8	50.8	52.0	60.9	44.8	44.4	47.6	56.0
BPG	33.6	39.8	40.2	46.2	43.3	44.8	34.4	43.4
J2K	37.2	48.0	39.6	54.9	44.4	49.1	37.2	53.7
JPEG	36.0	58.1	35.4	61.9	39.7	48.5	31.2	57.6
HEVC	26.7	36.6	36.2	45.7	37.1	41.3	31.1	40.4
VCC	29.1	36.1	33.7	47.5	37.5	43.1	30.1	42.2
AV1	29.8	39.0	32.9	47.0	38.4	44.8	30.0	42.4

**Table 6:** EERs for the different datasets using the CAHT algorithm.

Dataset	Casia4i		Casia5a		IITD		Notredame	
Level	B	A	B	A	B	A	B	A
DSSLIC	1.2	1.0	21.1	21.2	1.4	1.8	29.9	29.9
EOIC	1.2	1.4	23.6	25.0	2.8	2.3	30.9	38.8
BPG	1.0	1.2	21.6	21.3	1.6	2.4	29.6	30.3
J2K	1.1	1.3	20.6	22.3	2.0	2.6	30.0	30.1
JPEG	1.2	2.8	20.6	26.1	1.9	2.5	29.9	32.4
HEVC	0.9	1.0	17.3	19.1	1.9	1.8	30.5	30.5
VCC	0.7	1.0	17.0	18.6	1.4	2.3	30.1	29.9
AV1	0.9	1.1	17.4	18.5	1.6	2.0	29.7	30.0

**Table 7:** EERs for the different datasets using manual masks.

Dataset	Casia4i		Casia5a		IITD		Notredame	
Level	B	A	B	A	B	A	A	B
DSSLIC	0.4	0.4	2.5	2.9	0.4	0.5	23.8	23.9
EOIC	0.5	0.7	3.5	6.7	0.5	0.6	24.3	39.8
BPG	0.4	0.6	2.9	3.9	0.4	0.5	23.8	23.9
J2K	0.4	0.6	2.7	5.1	0.4	0.5	23.8	24.0
JPEG	0.5	1.7	3.0	14.0	0.4	0.5	23.8	25.7
HEVC	0.4	0.5	2.9	3.9	0.4	0.5	24.2	23.9
VCC	0.5	0.5	2.8	4.1	0.4	0.5	23.9	24.0
AV1	0.5	0.5	2.8	3.8	0.5	0.5	24.0	24.2

**Table 8:** EERs for different the datasets using the Osiris algorithm.

Dataset	Casia4i		Casia5a		IITD		Notredame	
Level	B	A	B	A	B	A	A	B
DSSLIC	1.1	1.0	2.0	2.2	0.7	0.8	25.2	25.5
EOIC	0.9	0.9	2.8	5.2	0.5	0.4	25.3	26.1
BPG	0.9	1.0	2.0	2.5	0.3	0.3	26.9	26.4
J2K	0.8	0.9	2.0	3.1	0.4	0.7	25.7	25.1
JPEG	0.8	1.8	2.4	9.7	0.5	0.6	24.7	24.7
HEVC	0.7	0.9	1.9	2.6	0.8	0.8	25.7	25.8
VCC	0.9	0.9	2.0	2.4	0.9	1.2	25.7	25.5
AV1	0.9	0.9	1.9	2.2	1.2	1.2	25.8	25.9

## Data Availability

Not applicable.
